# Clinical and genetic spectrum of a large cohort of patients with δ-sarcoglycan muscular dystrophy

**DOI:** 10.1093/brain/awab301

**Published:** 2021-09-13

**Authors:** Jorge Alonso-Pérez, Lidia González-Quereda, Claudio Bruno, Chiara Panicucci, Afagh Alavi, Shahriar Nafissi, Yalda Nilipour, Edmar Zanoteli, Lucas Michielon de Augusto Isihi, Béla Melegh, Kinga Hadzsiev, Nuria Muelas, Juan J Vílchez, Mario Emilio Dourado, Naz Kadem, Gultekin Kutluk, Muhammad Umair, Muhammad Younus, Elena Pegorano, Luca Bello, Thomas O Crawford, Xavier Suárez-Calvet, Ana Töpf, Michela Guglieri, Chiara Marini-Bettolo, Pia Gallano, Volker Straub, Jordi Díaz-Manera

**Affiliations:** 1 Neuromuscular Diseases Unit, Department of Neurology, Hospital de la Santa Creu i Sant Pau, Universitat Autònoma de Barcelona, Department of Medicine, Barcelona 08041, Spain; 2 Genetics Department, IIB Sant Pau, Hospital de la Santa Creu i Sant Pau, Universitat Autònoma de Barcelona, Barcelona 08041, Spain; 3 Centro de Investigación Biomédica en Red en Enfermedades Raras (CIBERER), Spain; 4 Center of Translational and Experimental Myology, IRCSS Istituto Giannina Gaslini, Genova 16147, Italy; 5 Genetics Research Center, University of Social Welfare and Rehabilitation Sciences, Tehran 13871, Iran; 6 Department of Neurology, Neuromuscular Research Center, Shariati Hospital, Tehran University of Medical Sciences, Tehran 14117, Iran; 7 Pediatric Pathology Research Center, Research Institute for Children Health, Shahid Beheshti University of Medical Sciences, Tehran 14117, Iran; 8 Department of Neurology, Hospital das Clínicas HCFMUSP, Faculdade de Medicina da Universidade de São Paulo, São Paulo 05403, Brazil; 9 Department of Medical Genetics, and Szentagothai Research Center, University of Pecs, School of Medicine, Pecs 07522, Hungary; 10 Neuromuscular Diseases Unit, Neurology Department, Hospital Universitari I Politècnic La Fe, Neuromuscular Reference Centre, ERN-EURO-NMD, Valencia, 46026, Spain; 11 Neuromuscular and Ataxias Research Group, Instituto de Investigación Sanitaria La Fe, Valencia 46026, Spain; 12 Department of Integrative Medicine, Federal University of Rio Grande do Norte, Campus Universitário Lagoa Nova, 59012-300 Natal, RN, Brazil; 13 University of Health Sciences, Antalya Research and Training Hospital, Department of Paediatric Neurology, Antalya 07100, Turkey; 14 Medical Genomics Research Department, King Abdullah International Medical Research Center (KAIMRC), King Saud Bin Abdulaziz University for Health Sciences, Ministry of National Guard-Health Affairs (MNGHA), Riyadh 14611, Saudi Arabia; 15 Department of Life Sciences, School of Science, University of Management and Technology (UMT), Lahore 54770, Pakistan; 16 State Key Laboratory of Membrane Biology and Beijing Key Laboratory of Cardiometabolic Molecular Medicine, Institute of Molecular Medicine and Peking-Tsinghua Center for Life Sciences and PKU-IDG/McGovern Institute for Brain Research, Peking University, 100871 Beijing, China; 17 Department of Neuroscience, University of Padova, Padova 35112, Italy; 18 Department of Neurology, Johns Hopkins University School of Medicine, Baltimore, MD 21205, USA; 19 The John Walton Muscular Dystrophy Research Centre, Newcastle University and Newcastle Hospitals NHS Foundation Trust, Newcastle-upon-Tyne NE1 3BZ, UK

**Keywords:** muscular dystrophies, delta-sarcoglycan, SGCD, LGMD-R6/2F, registries

## Abstract

Sarcoglycanopathies include four subtypes of autosomal recessive limb-girdle muscular dystrophies (LGMDR3, LGMDR4, LGMDR5 and LGMDR6) that are caused, respectively, by mutations in the *SGCA*, *SGCB*, *SGCG* and *SGCD* genes. Delta-sarcoglycanopathy (LGMDR6) is the least frequent and is considered an ultra-rare disease. Our aim was to characterize the clinical and genetic spectrum of a large international cohort of LGMDR6 patients and to investigate whether or not genetic or protein expression data could predict a disease’s severity.

This is a retrospective study collecting demographic, genetic, clinical and histological data of patients with genetically confirmed LGMDR6 including protein expression data from muscle biopsies.

We contacted 128 paediatric and adult neuromuscular units around the world that reviewed genetic data of patients with a clinical diagnosis of a neuromuscular disorder. We identified 30 patients with a confirmed diagnosis of LGMDR6 of which 23 patients were included in this study. Eighty-seven per cent of the patients had consanguineous parents. Ninety-one per cent of the patients were symptomatic at the time of the analysis. Proximal muscle weakness of the upper and lower limbs was the most common presenting symptom. Distal muscle weakness was observed early over the course of the disease in 56.5% of the patients. Cardiac involvement was reported in five patients (21.7%) and four patients (17.4%) required non-invasive ventilation. Sixty per cent of patients were wheelchair-bound since early teens (median age of 12.0 years). Patients with absent expression of the sarcoglycan complex on muscle biopsy had a significant earlier onset of symptoms and an earlier age of loss of ambulation compared to patients with residual protein expression.

This study confirmed that delta-sarcoglycanopathy is an ultra-rare neuromuscular condition and described the clinical and molecular characteristics of the largest yet-reported collected cohort of patients. Our results showed that this is a very severe and quickly progressive disease characterized by generalized muscle weakness affecting predominantly proximal and distal muscles of the limbs. Similar to other forms of sarcoglycanopathies, the severity and rate of progressive weakness correlates inversely with the abundance of protein on muscle biopsy.

## Teaser

Alonso-Pérez *et al*. describe the largest cohort to date of patients with the ultra-rare neuromuscular disorder delta-sarcoglycanopathy, and show that the severity and rate of progressive weakness correlates inversely with the abundance of protein expression on muscle biopsy.

## Introduction

Limb-girdle muscular dystrophies (LGMD) are a heterogeneous group of genetic diseases that affect skeletal muscle causing progressive loss of muscle fibres leading to muscle weakness predominantly affecting the pelvic and shoulder girdle.^1^ More than 30 genes causing different types of LGMD have been described so far. Among them, sarcoglycanopathies are one of the most frequent forms especially when symptoms onset during childhood.^2-7^ There are four sarcoglycan genes (*SGCA*, *SGCB*, *SGCG* and *SGCD*) causing autosomal recessive LGMD (LGMDR3–6 previously known as LGMD2C, D, E and F). The frequency of each type of sarcoglycanopathy varies depending on the studied population, although LGMDR3/LGMD2D and LGMDR5/LGMD2C are the two most frequent forms.^8^

Delta-sarcoglycanopathy (LGMDR6/LGMD2F) is caused by recessive mutations in the *SGCD* gene and was originally described in 1996,^9–11^ and is thought to be the least common type of sarcoglycanopathy although the number of existing cases is not known. There are only a few reports describing the clinical features of single cases or short cohorts and therefore neither the clinical features nor the disease progression over time is well known.^12–14^

Sarcoglycanopathies (LGMDR3–6) are in general severe disorders characterized by weakness onset at first decade of life leading to loss of ambulation during adolescence or early adulthood. However, patients with a milder phenotype with onset of symptoms after the second decade of life, a slowly progressive course and still ambulant after the age of 50 or 60 years have also been described.^15–18^ We have recently observed that symptoms’ onset before the age of 10 years, protein expression in the muscle biopsy of <30% or mutations leading to absence of protein expression were independent risk factors associated with a more severe phenotype characterized by early loss of ambulation.^8^ However, it is not known whether these are also risk factors of quick progression in patients with LGMD-R6.

To answer these questions, we collected demographic, genetic, clinical and muscle protein data of a large international cohort of patients with mutations in *SGCD*. Our aims were to describe the main clinical and genetic features, investigate potential genotype–phenotype correlations and identify factors influencing the progression of the disease.

## Materials and methods

### Study design

This is an observational retrospective cross-sectional study reviewing clinical and genetic data of patients with confirmed pathogenic mutations in the *SGCD* gene. Anonymized data from clinical reports were collected in a survey and stored in a secure server in the Hospital de la Santa Creu i San Pau (HSCSP). The study was approved by the Ethics Committee of the HSCSP. To identify patients for the study, we used two different strategies. On the one hand we contacted 128 paediatric and/or adult hospitals from 38 countries. Only 11 neuromuscular centres from nine different countries had patients with delta-sarcoglycanopathy and agreed to participate in the study. On the other hand, we also contacted colleagues that lead next-generation sequencing (NGS) studies in large cohorts of neuromuscular disease patients without diagnosis. These studies actually included 9610 patients. None of these patients had mutations in the *SGCD* gene.

### Patient cohort

The inclusion criteria for the study were: (i) genetically confirmed diagnosis of delta-sarcoglycanopathy by identification of two heterozygous or one homozygous pathogenic mutation in the *SGCD* gene; and (ii) enough data available in the clinical records to answer ∼70% of the variables of the survey about disease onset and progression, presence or absence of cardiac and respiratory involvement and muscle function status at last clinical assessment.

### Data sources

Participating centres completed a survey for each of the patients included in the study. The survey collected demographic, clinical and genetic data of all patients. We also collected information about the muscle biopsy if performed, including morphological features and levels of protein expression measured by immunohistochemistry or immunofluorescence. Cardiac involvement was defined according to international guidelines based on the presence of left ventricular ejection fraction lower than 50% and/or fractional shortening lower than 25% and/or the existence of morphological abnormalities in the ventricular walls evaluated by echocardiography or the existence of cardiac conduction defects identified using ECG or Holter monitoring.^19,20^ The need for ventilatory support was also collected as well as the age at which it was started. A copy of the survey is provided in the Supplementary material.

Mutations in the *SGCD* gene were centrally reviewed by experienced geneticists from HSCSP (L.G.-Q. and P.G.) to confirm pathogenicity and predict protein expression. We considered nonsense, frameshift and canonical splice site mutations as non-protein producing since these mutations will cause a disruption of the reading frame or a considerable shortening of the transcript resulting in mRNA elimination by nonsense-mediated decay or aberrant protein degradation. A sequence-based prediction of impact the of the missense mutations on protein function and/or expression was performed using different computational approaches assessing sequence conservation; for example, SIFT^21^ and PANTHER-PSEP,^22^ as well as sequence and structural features such as PolyPhen-2^23^ and MutationTaster.^24,25^ Deep intronic variants were considered compatible with protein production since these variants even when resulting in abnormal splicing they usually produce a residual amount of wild-type transcript.^26,27^

### Statistical analysis

Quantitative variables were analysed using the Shapiro–Wilk test to verify the normal distribution. Comparison between the different subgroups of patients was performed using the Chi-squared test for categorical variables and the Student's *t*-test or Mann–Whitney test for quantitative variables. We used a Cox proportional hazard regression model for the analyses of time to wheelchair. *P* was considered significant if lower than 0.05. Hierarchical analysis and graphical representation as a heatmap of muscle strength measured using the MRC scale was performed using R software v.3.1.3 as previously described.^28^ Statistical analysis was performed by J.D.-M. and J.A.-P. using SPSS^®^ Statistics software v.21 from IBM^®^.

### Data availability

The data that support the findings of this study are available from the corresponding author on reasonable request.

## Results

### Patient cohort

We contacted 128 paediatric and adult neuromuscular units and/or neurology departments around the world and identified a total of 30 patients with a confirmed genetic diagnosis of LGMDR6. We excluded seven patients because clinical data were not available or updated. The 23 patients included were from nine different countries: Spain, UK, Italy, Hungary, Turkey, Iran, Pakistan, Brazil and Canada. Additionally, we also contacted international centres that have lead research projects using NGS to sequence large cohorts of patients with neuromuscular diseases.^4,29–34^ After reviewing the results of 9610 patients, no patients with delta-sarcoglicanopathy were found.

Among the 23 identified patients with confirmed diagnosis of LGMDR6, 10 were males and 13 were females from 18 different families. Consanguinity was present in 20 patients (87%) and 12 patients (52.2%) had another relative affected by the disease.

Most of the patients (*n* = 21, 91.3%) were symptomatic at the time of data collection. However, two patients were considered presymptomatic as they had neither symptoms nor signs of neuromuscular involvement on clinical examination. These two patients were 4 and 5 years old, respectively, and relatives of other affected patients. Among the 21 symptomatic patients, median age at onset of symptoms was 5.0 years (range: 2–24 years). Diagnostic delay, defined as the time from onset of symptoms to the confirmed genetic diagnosis of the disease range from 1 to 37 years (median: 6.5 years). Median diagnostic delay in patients with onset of symptoms before 2010, when genetic testing was done using Sanger sequencing was of 10.0 years (range: 4–37, *n* = 13), while diagnostic delay in patients with onset of symptoms after 2010, when NGS studies started to be used in the diagnosis process of LGMD was of 2.0 years (range: 0–7, *n* = 10). These differences were statistically significant (*P* = 0.001, Mann–Whitney test).

Table 1 shows the most common symptoms at onset. In summary, proximal lower limb weakness was the most frequent symptom seen in 12 of 21 patients. Five patients (23.8%) complained of muscle pain associated with muscle weakness at the onset of the disease. Seven out of the 21 symptomatic patients were ambulant at their last visit. Median age at loss of ambulation was 12.0 years (range 9–37). Median time from onset of symptoms to loss of ambulation was 7.0 years (range 4–10). We characterized disease progression based on the data collected from the clinical reports and observed that 18 patients (85.7%) were not able to run since a median age of 8.0 years old, 16 patients (76.2%) were not able to stand up from a chair since a median age of 9.5 years and nine patients (42.9%) needed aids to walk since a median age of 10.0 years (Table 1).

**Table 1:** Demographic and clinical features

	LGMDR6/2F
No. of patients	23
Sex, male/female	10/13
Consanguinity, *n* (%)	21 (87)
Age onset, median ± SD (range)	5.0 ± 6.8 (2–24)
Diagnostic delay (onset–genetic), median ± SD (range)	6.5 ± 7 (1–37)
Age at last evaluation, median ± SD (range)	17.0 ± 12.3 (4–50)
Evolution of the disease, years, median ± SD (range)	11.5 ± 8.9 (3–33)
Symptom onset, *n* (%)	
Proximal lower limb weakness	12 (57.1)
Proximal upper limb weakness	2 (9.5)
Gait disturbance	11 (52.4)
Muscle pain	5 (23.8)
Motor function, *n* (%); median age ± SD (range)	
Stop running	18 (85.7); 8.0 ± 6.8 (3–32)
Impossibility to stand from a chair	16 (76.2); 9.5 ± 7.3 (7–34)
Walking with aids	9 (42.8); 10.0 ± 1.8 (9–14)
Wheelchair-bound	14 (66.7); 12.0 ± 7.1 (9–37)
Cardiac involvement, *n* (%); median age ± SD (range)	5 (23.8); 13.0 ± 2.7 (11–17)
Respiratory support, *n* (%); median age ± SD (range)	4 (19.0); 20.5 ± 5.1 (13–24)
Death, *n* (%)	0 (0)

*n* = number of families; SD = standard deviation.

**Figure 1:**
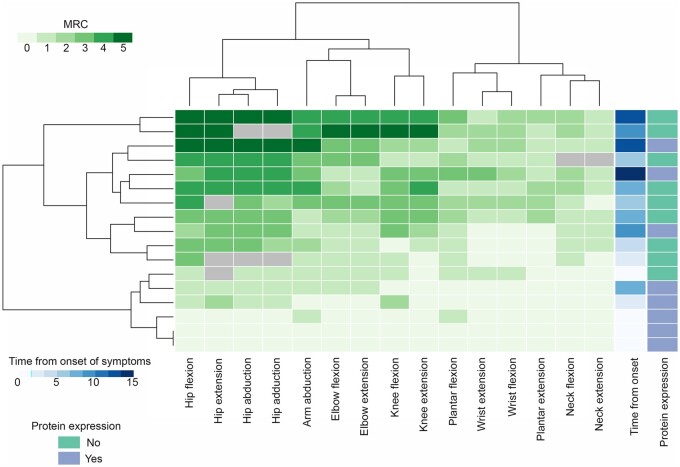
**Heat map showing an unsupervised hierarchical clustering of MRC values of patients included in the study.** The heat map shows the MRC value for all muscles studied. Patients and muscles were ordered automatically by the software in an unsupervised manner. Hip extension, flexion, abduction and adduction were the weakest movements followed by arm abduction, elbow flexion and extension, and knee flexion and extension. Muscle strength was measured using the MRC scale that scores muscle function from 0 to 5. We observed a correlation between the degree of muscle weakness and time from onset of symptoms and also between MRC and absence of protein expression on the muscle biopsy.

**Figure 2:**
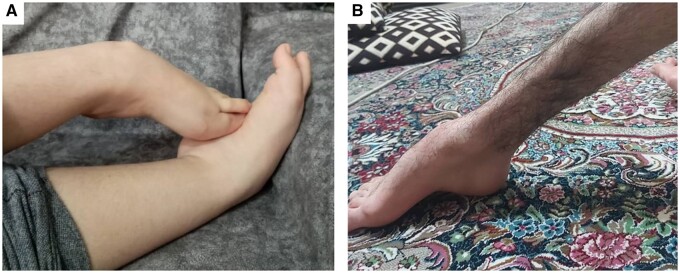
**Foot deformities.** We identified weakness of the distal muscles of the lower limbs in some of the patients in the cohort that lead to the frequent presentation of deformities as the two shown here. (**A**) A 9-year-old patient with bilateral clubfeet. (**B**) A pes cavus in an 18-year-old patient.

At last clinical evaluation, clinical examination showed proximal muscle weakness in all symptomatic patients. Axial (*n* = 13, 61.9%) and distal (*n* = 12, 57.1%) weakness was also present from early stages of the disease, especially in patients with no remaining protein expression (Fig. 1). Scoliosis was observed in 12 patients (57.1%) at a median age of 11.5 years (range 7–15). Scapular winging (*n* = 10, 47.6%), calf hypertrophy (*n* = 12, 57.1%) and generalized muscle atrophy (*n* = 13, 61.9%) were also described. Tiptoe walking due to Achilles tendon contractures was reported in 15 patients (71.4%) at a median age of 9.5 years (range 3–16). Foot deformities, mainly pes cavus due to early lower limb distal muscle weakness was also observed (Fig. 2).

Cardiac involvement, defined as cardiomyopathy or heart rhythm abnormalities, was reported in five patients (23.8%) with a median age of diagnosis of 13.0 years (range 11–17). Dilated cardiomyopathies were present in three patients (60%) and heart rhythm abnormalities were present in two patients. The last cardiac assessment showed median left ventricle ejection fraction of 40% (range 30–58). Four out of these five patients were under treatment because of the cardiac involvement. Median forced vital capacity at last assessment was of 62.0% (range 44–84). Four patients (19%) required ventilatory support from a median age of 20.5 years (range 13–24) and were using it a median of 9 h per night (range 6–15). None of the patients had a tracheostomy.

**Figure 3:**
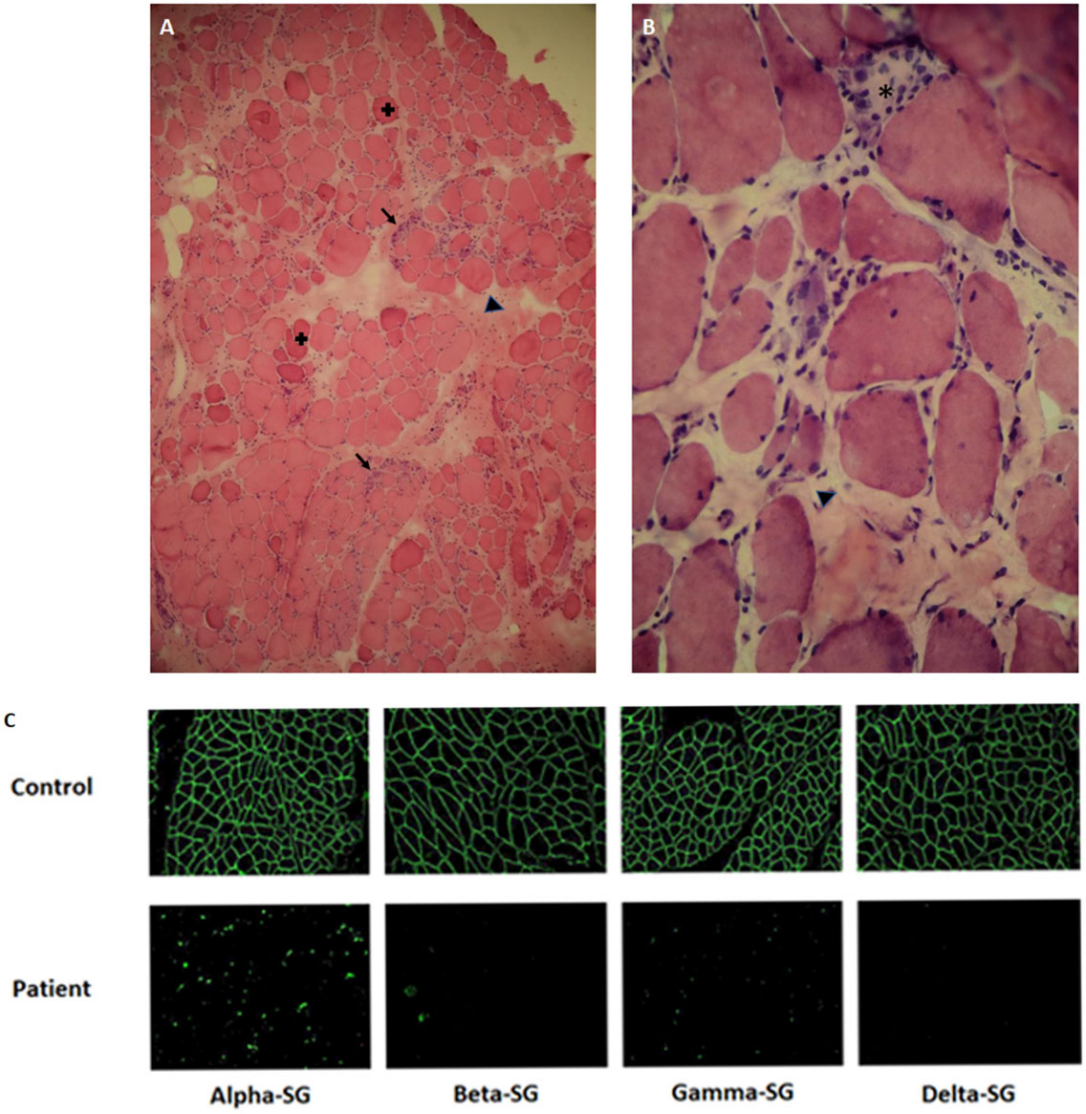
**Muscle biopsy.** (**A** and **B**) Haematoxylin-eosin staining. Necrotic fibres (asterisk), inflammatory infiltrate (arrow), increased fibrosis tissue (arrowhead), hypercontracted fibres (plus symbol). Magnification: ×20 (*left*) and ×40 (*right*). (**C**) Immunofluorescence of sarcoglycan subunits. SG = sarcoglycan.

A muscle biopsy was performed in 14 patients (60.9%) at a median age of 10.0 years (range 2–34) and a median time of disease duration of 4.5 years (range 1–30). Increase in the amount of fibrotic tissue and presence of necrotic muscle fibres were the most frequent features (64.3 and 50%, respectively). Inflammatory infiltrates were observed in 28.6% of biopsies (Fig. 3A and B). Residual expression of sarcoglycan proteins was studied using immunohistochemistry or immunofluorescence in all patients (Fig. 3C). Six patients had no protein expression of any sarcoglycan subunit while eight patients had some amount of residual expression. There were no differences in the age when biopsy was obtained (*P* = 0.295, Mann–Whitney test) or in the time of progression of the disease (*P* = 0.180, Mann–Whitney test) between patients with residual protein expression and those with no protein expression.

Table 2 shows the clinical, genetic, muscle biopsy features and protein expression of the patients included in this study.

**Table 2:** Clinical and genetic features c.699 + 1G>T

Pt	Mutation	Age at last evaluation	Age of onset	WCB, age	Distal weakness	Cardiopathy, age	NIV, age	Muscle biopsy	IHQ/IF	Protein expression
1	c.657delC, p.(Thr220Profs*6)	21	7	Yes, 15	Yes	No	No	No	↓↓	Yes
2	c.657delC, p.(Thr220Profs*6)	8	5	No	No	No	No	No	–	YES^a^
3	c.657delC, p.(Thr220Profs*6)	27	8	Yes, 15	Yes	No	No	Yes	↓↓	Yes
4	c.657delC, p.(Thr220Profs*6)	17	6	Yes, 10	Yes	No	No	Yes	↓↓	Yes
5	c.657delC, p.(Thr220Profs*6)	22	5	Yes, 14	Yes	Yes, 17	Yes, 18	Yes	↓↓	Yes
6	c.1_3del, p.(Met1del)	5	–	–	–	–	–	–	–	Yes^a^
7	c.1_3del, p.(Met1del)	25	23	No	No	No	No	No	–	Yes^a^
8	c.1_3del, p.(Met1del)	11	7	No	Yes	No	No	No	–	Yes^a^
9	c.1_3del, p.(Met1del)	37	24	No	No	No	No	Yes	↓	Yes
10	c.(3_4–52)_(187_193-1)del), p.(Met2_Ile64del)	5	3	No	No	No	No	Yes	↓↓	Yes
11	c.(3_4-52)_(187_193-1)del), p.(Met2_Ile64del)	4	–	–	–	–	–	Yes	↓↓	Yes
12	c.353-357del, p.(Thr119Serfs*17)	19	2	Yes, 11	Unknown	Yes, 12	No	Yes	Absent	No
13	c.353-357del, p.(Thr119Serfs*17)	24	9	Yes, 15	Yes	No	Yes, 23	Yes	Absent	No
14	c.568G>T, p.(Glu190*)	11	4	Yes, 9	Yes	No	No	No	–	No^a^
15	c.568G>T, p.(Glu190*)	16	5	Yes, 10	Yes	No	No	Yes	Absent	No
16	c.422dupT, p.(Thr143Asnfs*13)	10	5	No	Yes	No	No	No	–	No^a^
17	c.289C>T, p.(Arg97*)	13	3	Yes, 10	Yes	No	No	No	–	No^a^
18	c.248-249delCT, p.(Ser83*)	11	4	Yes, 10	Yes	Yes, unknown	No	Yes	Absent	No
19	c.89G>A, p.(Trp30*)	29	2	Yes, 9	Unknown	Yes, 19	Yes, 13	Yes	Absent	No
20	43	3	Yes, 12	Yes	Yes, 11	Yes, 24	Yes	Absent	No	
21	c.593G>C, p.(Arg198Pro)	–	4	Yes, 15	Unknown	Unknown	Unknown	Yes	↓↓	Yes
22	c.575+1G>T	50	22	Yes, 37	Unknown	Unknown	Unknown	Yes	↓↓	Yes
23	c.-519_502del), p.0	17	9	No	Yes	No	No	Yes	↓↓	Yes

IHQ/IF = Immunohistochemistry or immunofluorescence detection of protein expression of the whole sarcolgycan complex on muscle biopsies; NIV = non-invasive ventilation required and age at which was started; Pt = patient; WCB = age at which patients were wheelchair-bound. Patients 6 and 11 were asymptomatic at the last assessment.

a Identifies patients whose protein expression in the muscle was predicted.

### Genetics

**Figure 4:**
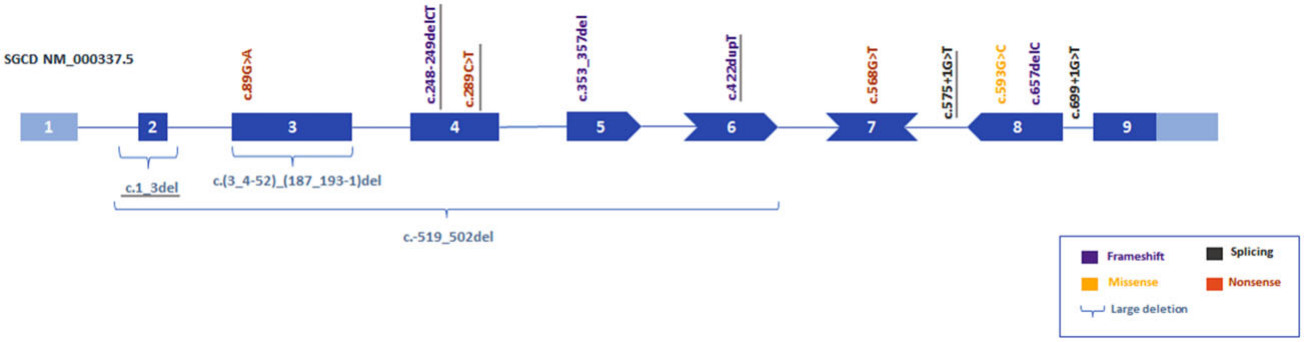
**Distribution of the pathogenic variants found in our cohort of patients in the *SGCD* gene.** The graph shows the distribution of the pathogenic variants identified in the patients that participated in the study. Novel variants not previously described are underlined. *Bottom right*: The legend describes the type of mutation.

Thirteen different pathogenic variants were identified in the *SGCD* gene (Fig. 4), six of them not previously described. All patients were homozygous for a single variant. The variants identified differed depending of the country of origin, with variant c.657delC, p.(Thr220Profs*6) being the most commonly detected in patients of Brazilian origin (21.7% of patients). Most of the pathogenic variants (65.2%) affected the extracellular domain of the protein. Four frameshift, three nonsense and two splicing mutations are reported in the present work, assuming that they will cause a premature termination of translation in case that the mRNA generated is not rapidly degraded by mRNA nonsense-mediated decay. In addition, three deletions are reported. The mutation c.1_3del;p.Met1del eliminates the translation initiation methionine (M1) located at the very end of exon 2. Protein production could still be possible since the first codon of exon 3 is also a methionine (M2) that could act as a starting point for the polypeptide, as is the case in the alternative transcript ENST00000435422.7. However, expression of the whole sarcoglycan complex in the muscle biopsy of the cases harbouring this mutation was identified (Table 2). The deletion c.4_192del eliminates 63 amino acids (p.Met2_Ile64del) yet keeps the reading frame so it is difficult to predict its effect on protein structure. This mutation was present in two patients of the cohort with a muscle biopsy that showed severe reduction in protein expression. The deletion c.-519_502del from exon 1 to 6 removes first half of the protein and all protein domains. We identified one missense mutation, p.(Arg198Pro) that has been previously reported as pathogenic.^35^ Assessing the pathogenic effect of a missense variant requires an understanding of its impact on the gene expression and protein structure and function. Missense mutations can affect protein function not only by disrupting their structure and conformation but also influencing its interaction with other proteins or molecules. Thus, the effect of missense mutations is not always easy to predict. This missense variant was neither found in gnomAD nor the 1000 Genomes databases, and its pathogenicity is supported by different prediction tools’ scores (Supplementary Table 1).

### Genotype–protein expression–phenotype correlation

To study the potential genotype–phenotype correlation in delta-sarcoglycanopathies, we studied the impact of the mutations on the residual protein expression in 14 muscle biopsies. Based on these data we estimated the residual protein expression in the nine remaining patients without muscle biopsy but who shared the same variants in the *SGCD* gene with the patients who had a muscle biopsy. Patients were classified in two groups: (i) no protein expression if patients had an undetectable expression of the sarcoglycan complex measured by muscle immunohistochemistry or immunofluorescence or carried two frameshift or nonsense mutations; and (ii) residual protein expression detected by immunohistochemistry or immunofluorescence.

We did not observe differences in the duration of the disease, defined as the time from onset of symptoms to last clinical evaluation, between both groups of patients (*P* = 0.49, Student’s *t-*test).

**Figure 5:**
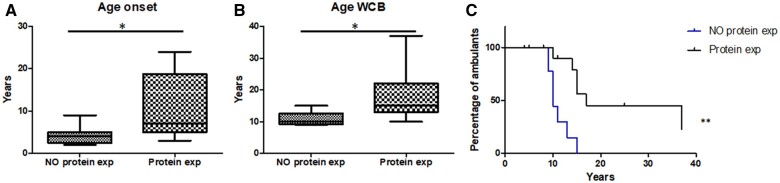
**Influence of remaining protein expression.** (**A**) Age of onset of patients with residual protein expression and patient with no protein expression. (**B**) Age of loss of ambulation of patients with residual protein expression and patient with no protein expression. Mann–Whitney test, *P < 0.05. (**C**) Kaplan–Meier estimates influence of remaining protein expression in age at wheelchair for patients with residual protein expression and patient with no protein expression. Mantel–Cox, **P < 0.001. exp = expression; WCB = wheelchair-bound.

Patients with no protein expression had an earlier disease onset (4.1 versus 10.3 years; *P* = 0.01, Mann–Whitney test) (Fig. 5A) and lost ambulation earlier (10.9 versus 17.3 years; *P* = 0.03, Mann–Whitney test) than patients with residual protein expression (Fig. 5B). Consequently, patients with residual protein expression maintained ambulation for a longer period of time (*P* = 0.001, Mantel–Cox test) (Fig. 5C). The correlation between the age of onset and progression of the disease remains if we analyse the data independently, depending on whether the protein level was obtained from muscle biopsy or by prediction (data not shown).

There were not significant differences in the prevalence of cardiac involvement (Chi-squared test, *P* = 0.09) or the needed for respiratory support (Chi-squared test, *P* = 0.21) between both groups.

## Discussion

We report the clinical, genetic and natural history data of the largest series of patients with delta-sarcoglycanopathy described so far. International collaboration has been crucial to gather this number of patients and to obtain information about clinical features and prognosis of the disease. As described in other types of sarcoglycanopathy, patients with residual protein expression had a later onset of disease symptoms and a milder clinical course characterized by later loss of ambulation than patients with no remaining protein expression. These data are especially relevant at present, as genetic therapies are under development and interventional clinical trials in patients with other types of sarcoglycanopathy are being designed.^36–38^

Sarcoglycanopathies, as a group, are one of the most common recessive LGMD^6,39,40^; however, delta-sarcoglycanopathy (LGMDR6) was supposed to be a very rare disease. In recent years, a series of studies have analysed large cohorts of patients with undiagnosed genetic muscle diseases using genes panels and/or whole exome sequencing. The frequency of LGMDR6 in these studies is very low or absent.^3,8,34,40^ Furthermore, published LGMDR6 reports so far have just included a few cases or isolated patients.^12,13,41^ A recent publication analysing a large cohort of European sarcoglycanopathies showed that only 1.5% of the cases were delta-sarcoglycanopathy.^8^ This previous evidence confirms that delta-sarcoglycanopathy can be considered an ultra-rare disease that it is defined as having a prevalence lower than 20 patients per million of population or one case per 50 000 habitants. In the case of delta-sarcoglycanopathy, the prevalence could be much lower probably in the order of one case per million of habitants. We do not have a clear explanation why patients with delta-sarcoglycanopathy are so infrequent compared to patients with other forms of sarcoglycanopathy. One potential explanation could be that mutations in this gene may be lethal during foetal development. If that were the case, this could be more probably related to a cardiac problem than to a skeletal muscle problem as there are no patients with congenital myopathy described so far. On the contrary, it is well known that mutations in the *SGCD* gene can be associated to developmental problems of the heart in preclinical animal models, such as zebrafish, and also to progressive cardiomyopathy both in humans and animal models.^42–45^ From a functional point of view, both the beta and the delta subunits are the core of the sarcoglycan complex suggesting than lack of beta-sarcoglycan should be as deleterious as the delta subunit for development, but patients with mutations in the *SGCB* are much more frequent than patients with mutations in the *SGCD* gene.^46–48^ Another reason could be that *SCGD* is devoid of sequence more prone to mutate compared to other sarcoglycan genes, but we observed that mutations in the *SGCD* were located in all the exons, same as the other sarcoglycan genes, suggesting that no particular exons are lethal if mutated.^8^

LGMDs produced by mutations in *SGCA*, *SGCB* and *SGCG* genes have been classically associated with a rapid progression of muscle weakness leading to severe disability, loss of ambulation in the second decade of life and frequently presenting cardiac and respiratory involvement.^8,49^ However, disease progression can be more heterogeneous as several patients, especially those with mutations in the *SGCA* gene, have a later onset of the disease and can loss ambulation later in life or even remain ambulant.^8,16,18^ To broaden the clinical presentation, patients with alpha-sarcoglycanopathy and isolated hyperCKaemia have been described.^50^ In contrast, patients with beta and gamma sarcoglycanopathies have in general a more homogeneous clinical picture with an early loss of ambulation.^8,18,49,51^ Our data shows that delta-sarcoglycanopathy is similar to other sarcoglycanopathies, with early onset in the first decade of life, rapid progression and loss of ambulation in the second decade of life in a high percentage of cases. However, we have observed that residual protein expression could influence clinical progression and severity of the disease. Indeed, 77.8% of patients with no remaining protein expression and 87.5% of patients with symptoms onset before the age of 5 years were wheelchair-bound at the age of 15 years. On the other hand, 21.4% of patients with residual protein expression and 25% of patients with symptom onset after the age of 5 years lose ambulation before adolescence. Moreover, only 42.9% of patients with residual protein expression ended up in a wheelchair and in all cases after the age of 10 years. These data support that residual protein expression is associated with a later onset of the disease and better prognosis as is shown in Fig. 3. This finding was also described for alpha, beta and gamma sarcoglycanopathies. However, despite the fact that patients with residual protein expression can have a milder disease progression, this disease is extremely severe as 91.3% of the patients were wheelchair-bound before the age of 18 years.

Beta and delta-sarcoglycan subunits form the core of the sarcoglycan complex and therefore are essential for its assembly.^47^ Mutations in the *SGCD* gene should therefore translate into a complete disruption of the complex leading to absence of expression of all sarcoglycan subunits in the muscle membrane and, consequently, to a more fragile membrane suffering more damage after every contraction, as has been described in the murine model of the disease.^48,52^ In fact, the delta-sarcoglycan murine model is characterized by severe muscle weakness and cardiac involvement.^13,53,54^ However, in this cohort some of the patients for whom a muscle biopsy was available (8 of 14) did have residual protein expression of the sarcoglycan subunits identified by immunohistochemistry. These patients presented with a later onset and less severe disease, suggesting that residual expression of the different components of the complex was enough to produce a better prognosis.

All patients were homozygous for one pathogenic variant and 87% had a history of consanguinity in the family. The most frequent types of mutation were frameshift mutations (up to 39.1% of the patients); however, 30.4% of patients carried a large deletion affecting one or more exons, which might be missed by Sanger sequencing and could only be confirmed by a quantitative technique such as multiplex ligation-dependent probe amplification (MLPA). It is therefore important to use MLPA in those cases with a high suspicion of the disease based on clinical and/or muscle biopsy data if Sanger sequencing does not identify any pathogenic mutation.^55,56^

Cardiac and respiratory involvement requiring ventilatory support is frequent in patients with sarcoglycanopathy.^8,57,58^ However, there are differences in the frequency and the type of cardiac or respiratory involvement depending on the mutated gene. For example, it is known that cardiac involvement in the form of a cardiomyopathy is more frequent in patients with beta-sarcoglycanopathy, than in patients with alpha or gamma-sarcoglycanopathy.^8,49,57,59^ Loss of delta-sarcoglycan is associated with cardiomyopathy is several preclinical animal models. For example, the different BIO14.6 hamster strains that harbour a deletion in the *SCGD* gene can develop either dilated or hypertrophic cardiomyopathy.^60^ In mice, delta-sarcoglycan deficiency leads to cardiomyopathy that is aggravated as a result of coronary artery vascular irregularities probably as a consequence of the absence of delta-sarcoglycan in the smooth muscle.^61^ Delta-sarcoglycan is key for normal cardiac development in zebrafish, leading to left–right asymmetry of the heart and disorganization of the intracellular myofibrils.^42,62^ There has been a long discussion related with the existence of isolated dominant cardiomyopathy produced by heterozygous mutations in the *SGCD* gene. Tsubata *et al*.^63^ found a single missense mutation (c.451T>G, p.Ser151Ala) associated with severe dominant cardiomyopathy in a family with three patients carrying this mutation. However, the pathogenic relevance of the p.Ser151Ala variant was challenged when this mutation was discovered in a large consanguineous family homozygous for p.Ala131Pro suffering from LGMDR6/2F but lacking any signs of cardiac disease in family members carrying the p.Ser151Ala mutation.^64^ Additionally, the knock-in of p.Ser151Ala caused a rather mild phenotype of cardiomyopathy in mice.^45^ Based on these data, the pathogenic potential of the p.Ser151Ala missense mutation to cause familial cardiomyopathy is unlikely. Surprisingly, we have only observed cardiac involvement in five patients in our cohort (23.8%), which was not related to a longer disease duration or with the level of residual protein expression. None of the patients had previous history of coronary heart disease. These data indicate that, in humans, delta-sarcoglycan defiency is not always associated to cardiac problems, although a longer follow-up of the patients included here is needed to confirm that they do not develop cardiac problems with age. Respiratory insufficiency requiring ventilatory support was only observed in four patients (19.0%) and was also not related with a longer disease duration or remaining protein expression. These data confirm that periodic assessment of cardiac and respiratory involvement is needed in all cases regardless of a patient’s age and the clinical status.

Our study has some limitations. First, data were collected retrospectively and there were some missing data in all cases. Second, quantification of protein expression was carried out through immunofluorescence performed at each centre with different antibodies and techniques including immunohistochemistry or immunofluorescence. Quantification of protein expression using western blot would have been better and could have helped us to identify a cut-off point predicting a more severe progression, as has been described in other types of sarcoglycanopathy.^8^ Moreover, for some patients, protein expression was estimated based on the results observed in patients sharing the same mutations in whom protein expression was studied in muscle biopsy. Although it is probable that protein expression is similar between patients with the same mutations, this is speculative and therefore these results should be interpreted cautiously. Despite our effort in contacting as many neuromuscular units as possible, we could have missed some delta-sarcoglycanopathy patients for inclusion into our study. An international registry for patients with mutations in the sarcoglycan genes, specifically in this case for patients with mutations in the *SGCD* gene could be useful to identify more patients. However, our series is the largest described so far and includes patients from different countries and ethnicities although there were no patients from Africa or South-East Asia.

In conclusion, our study provides new and relevant information that widens the knowledge on the clinical and genetic features of patients with mutations in the *SGCD* gene. We have identified that residual protein expression is associated with a later onset of disease and milder phenotype with later loss of ambulation. These data should be useful for the design of natural history studies and for clinical trial design, including gene replacement therapies, that are currently under development for other types of sarcoglycanopathy.

## Funding

This investigation was sponsored by a grant from the Spanish Ministry of Health, Fondos FEDER-ISCIII PI18/01525 to J.D.-M. J.A.P. was supported by the ‘Rio Hortega’ grant (CM19/00178), Acción Estratégica de Salud (EAS), Instituto de Salud Carlos III (Spain). B.M. and K.H. were supported by grants NKFIH 119540 and EFOP-3.6.1–16-2016–00004. L.G.-Q. and P.G. received funding from FIS PI8/01585, funded by ISCIII and FEDER, ‘Una manera de hacer Europa’.

## Competing interests

All authors report no competing interests.

## Supplementary material

Supplementary material is available at *Brain* online.

## Supplementary Material

awab301_Supplementary_Data

## Abbreviations

LGMD: limb-girdle muscular dystrophies
