# Supplementary material for: Clinical and genetic spectrum of a large cohort of patients with δ-sarcoglycan muscular dystrophy
Source: Brain. 2021 Sep 13;145(2):596–606. doi: 10.1093/brain/awab301 (PMC9014751; doi:10.1093/brain/awab301)
Supplement: awab301_Supplementary_Data [file awab301_supplementary_data.zip › brain-2021-01021-File009.pdf]

Supplemental table 1: Prediction of pathogeny of mutations identified in the cohort.

cDNA (Chr5(GRCh37), NM_000337.5	Protein	N	Mutation type	CADD	REVEL	DANN	Mutation Taster	Polyphen-2
c.657delC	p.Thr220Profs*6	5	Frameshift	.	.	.	.	
c.1_3del	p.Met1del	4	Frameshift deletion	.	.	.	.	
c.4_192del	p.Met2_Ile64del	2	In frame	.	.	.	.	.
c.568G>T	p.Glu190*	2	Nonsense	51	.	0.995	A	.
c.354_358del	p.Thr119Serfs*17	1	Frameshift	.	.	.	.	.
c.593G>C	p.Arg198Pro	1	Missense	35	0.953	0.998	D	
c.289C>T	p.Arg97*	1	Nonsene	41	.	0.998	D	0.999
c.89G>A	p.Trp30*	1	Nonsense	39	.	0.996	A	
c.575+1G>T	p.?	1	Splicing	25.9	.	0.995	D	
c.422dup	p.Thr143Asnfs*13	1	Frameshift	.	.	.	.	
c.248_249del	p.Ser83*	1	Nonsense	.	.	.	.	.
c.699+1G>T	p.?	1	Splicing	26.2	0.556	0.996	D	
c.-519_502del	p.?	1	Frameshift					

The table displays all variants identified in the patients of the cohort in the *SGCD* gene and their expected effect on protein as well as the number of patients harbouring the mutation (N), the mutation type and the results of the pathogenicity scores using the following prediction in Silico tools CADD, REVEL, DANN, Mutation Taster and Polyphen-2. For CADD a value higher than 20 is considered deleterious. In REVEL, scores higher than 0.5 are considered “likely disease causing”. Using DANN scores ranges from 0 to 1, the higher the value the more likely the mutation to be deleterious. Mutation Taster provides 5 categories being D: disease causing, A: disease causing automatic, N: polymorphism and P: polymorphism automatic. Using Polyphen-2 values greater than 0.908 are considered “probably damaging”.
