# Supplementary material for: Clinical and genetic spectrum of a large cohort of patients with δ-sarcoglycan muscular dystrophy
Source: Brain. 2021 Sep 13;145(2):596–606. doi: 10.1093/brain/awab301 (PMC9014751; doi:10.1093/brain/awab301)
Supplement: awab301_Supplementary_Data [file awab301_supplementary_data.zip › brain-2021-01021-File010.pdf]

Center ________________

Patient Initials ________________ Date of birth ________________

Sex M F

Ethnicity ________________

Age at last visit: _________ years

Genetics

Gene Mutated ☐ SGCD

Consanguinity ☐ No ☐ Yes

Mutation 1: c.__________________ mutation 2: c.__________________

p.__________________ p.__________________

Laboratory that performed the analysis: ____

Date of the analysis: __________________

Other family member affected: ☐ No ☐ Yes

Other family member included in the study: ☐ No ☐ Yes

Family tree (if necessary):

Muscle biopsy

Age at biopsy ________________ disease duration * ________________

** Years since the appearance of first reported symptoms*

Muscle biopsied: ☐ Quadriceps ☐ Deltoid ☐ Other ________________

H&E: Brief description

☐ Fibrotic ☐ Central nuclei >4 %
☐ Cell necrosis ☐ Ring fibers
☐ fiber variability ☐ Inflammation
☐ Other ____
____

Other staining ____________ ____
____________ ____

**Muscle biopsy: Western Blot**	Absent	<30% of control	30-60% of controls	Normal	Not performed
Alpha-Sarcoglycan	☐	☐	☐	☐	☐
Beta-Sarcoglycan	☐	☐	☐	☐	☐
Gamma-Sarcoglycan	☐	☐	☐	☐	☐
Delta-Sarcoglycan	☐	☐	☐	☐	☐
Other membrane proteins:					
Exact WB percent of control:					
	☐	☐	☐	☐	☐
	☐	☐	☐	☐	☐

**Muscle biopsy: IF**	Absent	Markedly reduced	Slightly reduced	Normal	Not performed
Alpha-Sarcoglycan	☐	☐	☐	☐	☐
Beta-Sarcoglycan	☐	☐	☐	☐	☐
Gamma-Sarcoglycan	☐	☐	☐	☐	☐
Delta-Sarcoglycan	☐	☐	☐	☐	☐
Other membrane proteins:					
	☐	☐	☐	☐	☐
	☐	☐	☐	☐	☐

Natural History

Age at first symptoms: ______Years ☐ Still asymptomatic ☐ Unknown

Type of onset: ☐ Slowly progressing ☐ Subacute ☐ Acute

First symptoms:

- ☐ Difficulty in running
- ☐ Difficulty in getting up from floor
- ☐ Difficulties in climbing stairs
- ☐ Gait difficulties
- ☐ Other: ___________________ ___________________
- ☐ Frequent falls
- ☐ Muscle pain
- ☐ Proximal weakness of upper limbs
- ☐ Respiratory symptoms

___________________ ___________________

Age at independent ambulation: ___________ months ☐ Unknown

Motor Function Evolution				
	Yes	*If yes: age*	***No***	***Unknown***
Stop **running**	☐		☐	☐
Impossibility to stand from a **chair**	☐		☐	☐
Use of walking aids (**cane**)	☐		☐	☐
Stop climbing **stairs**	☐		☐	☐
Loss of ambulation (**wheelchair**)	☐		☐	☐
**Bed-bound**	☐		☐	☐
**Respiratory support**	☐		☐	☐
Diagnosis of **cardiomyopathy**	☐		☐	☐
**Death**	☐		☐	☐

Cause of death:
- ☐ cardiac pathology (specify) __________________________
- ☐ respiratory pathology (specify) __________________________
- ☐ other (specify) __________________________

Motor Function: signs and symptoms

Actual best motor function:
- ☐ Running
- ☐ Walking without aids
- ☐ Walking with aids
- ☐ Wheel-chair bound
- ☐ Bed-bound

Tendon contraction: ☐ unknown ☐ no ☐ yes Achilles age ________
Rigid spine age ________
Other: _________ age ________
_________ age ________
_________ age ________

Tiptoe gait pattern:	☐Unknown	☐ No	☐ Yes	age ________
Calf hypertrophy	☐Unknown	☐ No	☐ Yes	age ________
Muscle Pain	☐Unknown	☐ No	☐ Yes	age ________
Scoliosis:	☐Unknown	☐ No	☐ Yes	age ________
Scapular winging:	☐Unknown	☐ No	☐ Yes	age ________
Macroglossia:	☐Unknown	☐ No	☐ Yes	age ________

Hearing loss: ☐ Unknown ☐ No ☐Yes ☐ Monolateral age ________
☐ Bilateral

Dysphagia: ☐ Unknown ☐ No ☐ Yes age ________

Other symptoms/signs: ________________________ age ________
________________________ age ________
________________________ age ________
________________________ age ________

ASSOCIATED PATHOLOGIES

____________________________________ age ________
____________________________________ age ________
____________________________________ age ________
____________________________________ age ________
____________________________________ age ________
____________________________________ age ________
____________________________________ age ________

Drug treatment for muscular function:

☐No ☐ Yes (specify: __________________________________ (age ______)
__________________________________ (age ______)
__________________________________ (age ______)

Muscle Strength (MRC)

Year of evaluation	
Age	
Disease duration	

	**R**	**L**
Shoulder abduction (deltoid)		
Elbow flexion (biceps)		
Elbow extension (triceps)		
Wrist flexion		
Wrist extension		
Hip flexion (psoas)		
Hip extension (gluteus maximus)		
Hip adduction		
Hip abduction		
Knee flexion (hamstrings)		
Knee extension (quadriceps)		
Ankle dorsiflexion (tibialis ant)		
Ankle plantar flexion (gastrocn)		
Neck flexion		
Neck extension		
Axial muscles		
Facial weakness		

Six-Minute Walk test: ______________ **m** age ________

☐ with walking aids ☐ Without Walking aids

Cardiac function

Cardiac symptoms no ☐ yes ☐

If yes, please answer the following questions:

- Dyspnoea: unknown ☐ no ☐ yes ☐ age ________
- Oedema unknown ☐ no ☐ yes ☐ age ________

- Palpitations unknown ☐ no ☐ yes ☐ age ________
- Other: ______________ age ________
______________ age ________

Cardiovascular disease no ☐ yes ☐

If yes, please answer the following questions:

- Atrial fibrillation: unknown ☐ no ☐ yes ☐ age ________

- Other heart rhythm abnormalities:
unknown ☐ no ☐ yes ☐ age ________
Specify: __________________

- Cardiomyopathy: unknown ☐ no ☐ yes ☐ age ________
If yes, *specify*:
☐ Dilated
☐ Hypertrophic
☐ Wall motion abnormalities
☐ Other ______________________________

LVEF: ___________% age ________

- Myocardial infarction: unknown ☐ no ☐ yes ☐ age ________
heart region: __________________

- Other: ____ age ________
____ age ________

Devices (ICD, etc) unknown ☐ no ☐ yes ☐ age ________
Type of device_______________

Cardiac drug treatment: no ☐ yes ☐

If yes, please answer the following questions:

Drug____________ indication_____________ dose_____________age at start_________

Drug____________ indication_____________ dose_____________age at start_________

Drug____________ indication_____________ dose_____________age at start_________

Respiratory Function

Respiratory symptoms: no ☐ yes ☐

If yes, please answer the following questions:

Respiratory symptoms:

Effort dyspnoea	unknown ☐	no ☐	yes ☐	age ________
Dyspnoea at rest	unknown ☐	no ☐	yes ☐	age ________
Morning headache	unknown ☐	no ☐	yes ☐	age ________
Recurrent pneumonia:	unknown ☐	no ☐	yes ☐	age ________
Other:	________________________________			age ________
	________________________________			age ________
	________________________________			age ________

Forced vital capacity (sitting): ________ l ; ________ % age ________

**Non invasive mechanical ventilation**	no ☐	yes ☐	age ________	hours/day _____
**Invasive mechanical ventilation**	no ☐	yes ☐	age ________	hours/day _____
Oxygen supplementation	no ☐	yes ☐	age ________	hours/day _____
Temporary tracheotomy	no ☐	yes ☐	age ________	

Hospitalizations for respiratory insufficiency (pneumonia): no ☐ yes ☐

Number of hospitaliz.during last 10 years ____________

Notes and Other
